# Pricing strategies for new product and remanufactured product considering emission sensitive demand

**DOI:** 10.1371/journal.pone.0288225

**Published:** 2023-09-06

**Authors:** Xixi Huang, Zhenkai Lou, Xiaozhen Dai, Lieying Luo

**Affiliations:** 1 School of Safety Management, Zhejiang College of Security Technology, Zhejiang, China; 2 School of Management Science and Engineering, Anhui University of Technology, Anhui, China; 3 School of Management, Wenzhou Business College, Zhejiang, China; West Pomeranian University of Technology, POLAND

## Abstract

With the rapidly increasing concern on environmental pollution and resource shortage, remanufactured products attract many attentions. In order to determine the optimal production and pricing strategy, we construct decision models for both single-product market and mixed-product market. Consumers’ different preferences for new products and remanufactured products are considered. First, we construct pricing models for a single-product market, and achieve a judging condition to determine the optimal strategy. Second, we develop a pricing model for a multiple-product market and put forward a suppose to show that the multiple-product strategy is not always optimal. Finally, numerical illustrations are designed to examine the impacts of the two crucial factors and obtain the dominant regions for each strategy. By introducing an emission sensitive demand, we show the superiority of the remanufactured product when the extra demand attracted by the emission saving is large.

## Introduction

Recent years, problems of environmental pollution and resource scarcity have attracted more and more attentions. Governments and enterprises are within their research to make different efforts on these aspects. In practice, governments formulate proper carbon tax policies to restrain the production of products with high pollution and encourage the production of green products [1, 2]. A higher carbon tax rate is proved to be beneficial from an environmental point of view [3]. As is well known, promoting the development of closed-loop supply chains is an effective approach to enhance the yield of green products.

With the raising of consumers’ environmental awareness, the green segment is becoming increasingly important to firms [4]. In Europe, 83% of consumers pay attention to the impact of products over environment [5]. Upwards of 60% of respondents are willing to pay such a premium for green products, on average, the premium that consumers are willing to pay is 16.8% [6]. Following these viewpoints, this study thinks that the emission saving of the remanufactured product attracts extra consumers.

Different from traditional supply chains, closed-loop supply chains focus on recycling and remanufacturing so as to lower the waste of resource, which is regarded as an environmentally friendly and profitable pattern [7]. In many cases, the remanufactured product has the same performance as the new product [8], and we also hold this opinion in this study. Apparently, how to recycle the core component plays a critical rule in the remanufacturing process.

This study considers a manufacturer who produces and sells new products and remanufactured products strategically. Consumers’ different preferences are taken into consideration. For some traditional consumers, they prefer new products rather than remanufactured products. For other consumers who concern carbon emission, the emission saving will attract their attentions. An emission saving is generated when producing a remanufactured product. We aim to examine both single-product market and mixed-product market so as to obtain the optimal strategy under a certain circumstance.

Remanufacturing is a recovery process that transforms a used product into a “like-new” product [9]. In practice, many factors are involved when considering the product remanufacturing, such as carbon tax, production strategy, recycling approach, etc. Carbon tax and production subsidy are two main means of governments to promote the production of remanufactured products. [10] analyzed the impacts of carbon taxes and carbon caps on the remanufacturing decisions, and proposed the optimal carbon policy for the considered scenario. [11] considered remanufacturing subsidy policy and carbon tax policy in a dual-channel supply chain selling both remanufactured and new products. [12] discussed a tax policy, a subsidy policy, and a tax-subsidy policy for a remanufactured problem, and designed a proper regulatory policy. [13] considered both fixed carbon emissions and variable carbon emissions for remanufacturing products, and presented three production decision models.

The production process of the remanufactured product is crucial for a manufacturer. [14] discussed a two-period manufacturing process, in which the remanufacturing activities happen at the second period. [15] constructed two-period production decision models in which the manufacturer produces new products in the first period and makes new and remanufactured products in the second period, aiming to acquire the optimal carbon emission tax policy. [16] considered pure manufacturing and hybrid manufacturing systems, and obtained production and sustainability level decisions by analyzing multiple settings. Recycling mode is another focus of manufacturers. This paper involves manufacturer recycling and third-party recycling. [17, 18] discussed cases in which manufacturers are in charge of recycling. [19, 20] examined the third-party-recycling mode for the remanufacturing.

Despite the abundant literature, there are still some research gaps. First, the consumer’s preference for emission saving hasn’t been explored thoroughly. Some potential consumers who care about carbon emission reduction may be attracted by the emission saving of the remanufactured product. Moreover, some traditional consumers may accept the remanufactured product as a substitution, but others may don’t. This paper pays attention to this phenomenon and examines the impacts of the preference. Second, a binary-product strategy may not be better than a single-product strategy. We aim to determine the dominant region for each strategy. Similar to [21], we focus on production and pricing strategies in this study. The sales quantities (or production quantities) of both the new product and the manufactured product are formulated as a linear function with respect to the unified sales price, following [22]. Table 1 compares the proposed model and the existing models.

**Table 1:** Summary of literature review.

Authors	Demand pattern	Emission sensitive demand	Low carbon policies
Gu et al. (2015)	Deterministic	No	No
Liu et al. (2015)	Stochastic	No	Carbon tax
Gan et al. (2017)	Deterministic	No	No
Wang et al. (2018)	Deterministic	No	Carbon tax
Cao et al. (2020)	Deterministic	No	Carbon tax
Ranjbar et al. (2020)	Deterministic	No	No
Ghosh et al. (2020)	Stochastic	Yes	Cap-and-trade
Ruidas et al. (2021)	Deterministic	No	Multiple policies
Our proposed model	Deterministic	Yes	Carbon tax

The remainder of this paper is organized as follows. In Section 2, we introduce the notations and make some assumptions for the given setting. Pricing models for a single-product market are constructed in Section 3. In Section 4, we propose a pricing model for a mixed-product market. Section 5 designs numerical illustrations to examine the sensitivity of the crucial parameters. Section 6 summarizes the study and shows the further research topics.

## Model description and assumption

This paper discusses production and pricing strategies of new product and remanufactured product in the presence of consumers’ different preferences. For some traditional consumers, they prefer new products rather than remanufactured products. For other consumers who concern carbon emissions, the emission saving per remanufactured product will induce their purchase. We consider two types of markets in this study, i.e., a single-product market and a binary-product market. The manufacturer involved in this study is meanwhile a seller, which means he produces products and then sells by himself.

The notations used in the following discussion are given by Table 2.

**Table 2:** Model parameters.

Parameters/variables	Description
Parameters	
*d*	The tradition demand which prefers new products
*β*	The ratio of the traditional demand that is willing to purchase remanufactured products
*δ*	The linear price-sensitive coefficient of the sales quantity
*θ*	The price substitution coefficient, showing the price competition level when the two types of products are sold simultaneously, 0 < *θ* < *δ*
*c*	The production cost of a new product
*s*	The cost saving of a remanufactured product. So, *c* − *s* is the production cost of a remanufactured product
*e*	The carbon emission of one new product
*∆e*	The emission saving of a remanufactured product. So, *e* − *∆e* is the carbon emission per remanufactured product
*λ*	The carbon tax per unit emission
*φ∆e*	The extra demand quantity that is attracted by the emission saving of the remanufactured product
Variables	
*p*_*n*_	The sales price of the new product
*p*_*r*_	The sales price of the remanufactured product
*q*_*n*_	The sales quantity of the new product
*q*_*r*_	The sales quantity of the remanufactured product

The models of this paper are mainly based on the following assumptions.

**Assumption 1.** *The tradition demand always tends to the new product*, *unless the remanufactured product is declared a lower price*.

**Assumption 2.** *The scale of the tradition demand quantity is large enough to guarantee that the demand is positive when the tradition demand quantity is the minuend*.

We make precise explanation for Assumption 2. According to [23], *d* − *δc* − *δλe* > 0 is a necessary condition to guarantee that the demand of the new product is positive in the single-product market. Actually, this condition always holds for almost all literatures with regard to pricing. Assumption 2 needs more stringent requirements, i.e.,

βd+φΔe−δ(c−s)−δλ(e−Δe)>0

and

d−(δ+θ)c−(δ+θ)λe>0.

If these conditions don’t hold, some sales models are infeasible. In the following discussion, we will show this judgment.

According to the above setting, we first show the demand quantity of each product when only one type of product is sold. The demand quantity of the new product is

$$q_{n}=d-\delta p_{n}$$

And the demand quantity of the remanufactured product is

$$q_{r}=\beta d-\delta p_{r}+\varphi \Delta e.$$

Further, the demand quantity of each type of product is formulated according to Assumption 1 when new products and remanufactured products are sold at the same time. The demand of a product decreases with the increase in retail price [24]. Following [25, 26], we adopt an item involving price spread to describe the cross influence on demand quantities. Thus, the demand quantity of the new product is formulated as

$$q_{n}=d-\delta p_{n}+\theta (p_{r}-p_{n}).$$

And the demand quantity of the remanufactured product is

$$q_{r}=\theta (p_{n}-p_{r})-\delta p_{r}+\varphi \Delta e.$$

In addition, we assume that the demand information is complete for the manufacturer, and the recyclable waste products are sufficient so as to guarantee that the manufacturer can produce enough remanufactured products to meet the consumers’ demand.

## Pricing models for a single-product market

In practice, multiple-product strategies are not always optimal. In this section, we consider issues that the manufacturer only produces and sells one type of product.

First, we consider the situation in which only new products are sold. The objective function of the manufacturer is

$$\pi_{n}=(d-\delta p_{n})(p_{n}-c-\lambda e).$$

This is a quadratic function with a negative quadratic term. According to the properties of quadratic functions, the solution of max *π*_*n*_ is (marking “*” at the top right corner, similarly hereinafter)

$$p_{n}=\frac{d+\delta c+\delta \lambda e}{2\delta }.$$ (1)

The demand quantity of the new product under (1) is

$$q_{n}=\frac{d-\delta c-\delta \lambda e}{2}.$$

According to Assumption 2, *q*_*n*_ > 0.

By substituting (1) into *π*_*n*_, we have

$$\pi_{n}=\frac{{\left(d-\delta c-\delta \lambda e\right)}^{2}}{4\delta }.$$ (2)

Second, we consider the situation in which only remanufactured products are sold. The objective function of the manufacturer is

$$\pi_{r}=(\beta d-\delta p_{r}+\varphi \Delta e)[p_{r}-(c-s)-\lambda (e-\Delta e)].$$

This is a quadratic function with a negative quadratic term. According to the properties of quadratic functions, the solution of max *π*_*r*_ is

$$p_{r}=\frac{\beta d+\varphi \Delta e-\delta (c-s)-\delta \lambda (e-\Delta e)}{2\delta }.$$ (3)

The demand quantity of the remanufactured product under (3) is

$$q_{r}=\frac{\beta d+\varphi \Delta e-\delta (c-s)-\delta \lambda (e-\Delta e)}{2}.$$

According to Assumption 2, *q*_*r*_ > 0.

By substituting (3) into *π*_*r*_, we have

$$\pi_{r}=\frac{{\left[\beta d+\varphi \Delta e-\delta (c-s)-\delta \lambda (e-\Delta e)\right]}^{2}}{4\delta }.$$ (4)

Profits gained by selling new products and remanufactured products are compared by (2) and (4). We have

$$\pi_{n}-\pi_{r}=\frac{\left[(1-\beta )d-\varphi \Delta e-\delta s-\delta \lambda \Delta e][(1+\beta )d+\varphi \Delta e-2\delta c+\delta s-2\lambda \delta e+\delta \lambda \Delta e\right]}{4\delta }.$$

Clearly, whether the value of (*π*_*n*_−*π*_*r*_) is positive or negative, depends on

(1−β)d−φΔe−δs−δλΔe. (5)

According to the above result, we draw the following conclusion:

**Proposition 1** In a single-product market: selling new product is better when

(1−β)d−φΔe−δs−δλΔe>0.

Otherwise, selling remanufactured product is better when

(1−β)d−φΔe−δs−δλΔe<0.

We expound the reasonability of Proposition 1 by making analysis for (5). Clearly, (1 − *β*)*d* denotes the decreased demand of remanufactured product, *φ*∆*e* denotes the increased demand of remanufactured product brought by carbon emission reduction, *δs* denotes the increased demand of remanufactured product brought by low production cost, and *δλ*∆*e* denotes the increased demand of remanufactured product brought by low carbon tax. Hence, when

(1−β)d>φΔe+δs+δλΔe,

the decreased demand of remanufactured product is larger than the increased demand, which means selling new product is better than selling remanufactured product.

## A pricing model for a mixed-product market

This section considers a mixed-product market, in which both new products and remanufactured products are sold. The price spread works. We will show the necessary condition for this mode to be practicable.

In this situation, the objective function of the manufacturer is

$$\pi =[d-\delta p_{n}+\theta (p_{r}-p_{n})](p_{n}-c-\lambda e)+[\theta (p_{n}-p_{r})-\delta p_{r}+\varphi \Delta e][p_{r}-(c-s)-\lambda (e-\Delta e)].$$

The following equation set is obtained by differentiating *π*:

$$\left\{ \begin{array}{l} \frac{\partial \pi }{\partial p_{n}}=-2(\delta +\theta )p_{n}+2\theta p_{r}+d+(\delta +\theta )(c+\lambda e)=0\\ \frac{\partial \pi }{\partial p_{r}}=-2(\delta +\theta )p_{r}+2\theta p_{n}+\varphi \Delta e+(\delta +\theta )[(c-s)+\lambda (e-\Delta e)]=0 \end{array}.\right.$$

By solving the above equation set, we have

$$\left\{ \begin{array}{l} p_{n}=\frac{\left(\delta +\theta )d+\theta \varphi \Delta e+{\left(\delta +\theta \right)}^{2}(c+\lambda e)+(\delta +\theta )\theta [(c-s)+\lambda (e-\Delta e)\right]}{2{\left(\delta +\theta \right)}^{2}-2\theta^{2}}\\ p_{r}=\frac{\theta d+(\delta +\theta )\varphi \Delta e+(\delta +\theta )\theta (c+\lambda e)+{\left(\delta +\theta \right)}^{2}[(c-s)+\lambda (e-\Delta e)]}{2{\left(\delta +\theta \right)}^{2}-2\theta^{2}} \end{array}.\right.$$ (6)

The Hessian matrix of *π* is

$$H=[\begin{array}{l} -2(\delta +\theta )\;2\theta \\ \;2\theta \;-2(\delta +\theta ) \end{array}].$$

Apparently, *H* is negative definite. Hence, the concavity of *π* is demonstrated, and the solution given by (6) is the solution of max *π*. Next, we examine the demand quantity of each type of product.

The demand quantity of the new product under (6) is

$$q_{n}=\frac{\left(\delta^{2}+2\delta \theta )[d-(\delta +\theta )(c+\lambda e)\right]}{2{\left(\delta +\theta \right)}^{2}-2\theta^{2}}.$$

According to Assumption 2, *q*_*n*_ > 0.

The demand quantity of the remanufactured product under (6) is

$$q_{r}=\frac{\left(\delta^{2}+2\delta \theta )\varphi \Delta e-(\delta^{3}+3\delta^{2}\theta +2\delta \theta^{2})[(c-s)+\lambda (e-\Delta e)\right]}{2{\left(\delta +\theta \right)}^{2}-2\theta^{2}}.$$

Clearly, whether *q*_*r*_ > 0 or not, depends on the value of

$$(\delta^{2}+2\delta \theta )\varphi \Delta e-(\delta^{3}+3\delta^{2}\theta +2\delta \theta^{2})[(c-s)+\lambda (e-\Delta e)].$$ (7)

According to [27], the manufacturer produces and sells one type of product when (7) is negative, which degenerates to the case proposed in Section 3. In order to maintain a mixed-product market, it is a necessary condition that (7) is positive. Actually, an equivalent form of *q*_*r*_ > 0 is

$$\frac{\varphi \Delta e}{2}-\frac{\left(\delta +\theta )[(c-s)+\lambda (e-\Delta e)\right]}{2}>0.$$ (8)

By (8), we have

$$\frac{\beta d+\varphi \Delta e-\delta (c-s)-\delta \lambda (e-\Delta e)}{2}>0,$$

which is one condition of Assumption 2. Hence, Assumption 2 is the premise and necessary condition of this discussion.

The profit of the manufacturer in a mixed-product market is

$$\begin{array}{l} \pi =\frac{(\delta^{2}+2\delta \theta )(\delta +\theta )d^{2}-2{\left(\delta^{2}+2\delta \theta \right)}^{2}(c+\lambda e)d+2(\delta^{2}+2\delta \theta )\theta \varphi \Delta ed}{{\left[2{\left(\delta +\theta \right)}^{2}-2\theta^{2}\right]}^{2}}\\ +\frac{2\delta \theta {\left(\delta +\theta \right)}^{2}[(c-s)+\lambda (e-\Delta e)]d+(\delta^{2}+2\delta \theta -\theta^{2})(\delta^{3}+3\delta^{2}\theta +2\delta \theta^{2}){\left(c+\lambda e\right)}^{2}}{{\left[2{\left(\delta +\theta \right)}^{2}-2\theta^{2}\right]}^{2}}\\ -\frac{2\delta \theta^{2}{\left(\delta +\theta \right)}^{2}(c+\lambda e)[(c-s)+\lambda (e-\Delta e)]+(\delta^{3}+\delta^{2}\theta )(\delta^{2}+2\delta \theta -\theta^{2}){\left[(c-s)+\lambda (e-\Delta e)\right]}^{2}}{{\left[2{\left(\delta +\theta \right)}^{2}-2\theta^{2}\right]}^{2}}\\ -\frac{(2\delta^{3}\theta +2\delta^{2}\theta^{2}-2\delta \theta^{3})\varphi [(c-s)+\lambda (e-\Delta e)]\Delta e-(\delta^{2}+2\delta \theta )(\delta +\theta )\varphi^{2}\Delta e^{2}}{{\left[2{\left(\delta +\theta \right)}^{2}-2\theta^{2}\right]}^{2}}. \end{array}$$

Clearly, it is hard to compare *π* with the profits gained in the single-product market *π*_*n*_ and *π*_*r*_. We present the following suppose, and verify it in the next section by numerical examples.

**Suppose 1** A multiple-product strategy is not always optimal.

Suppose 1 reveals the following fact: although a mixed-product market retains consumers that reject remanufactured products and meanwhile attracts extra consumers by the emission saving, the price competition between new products and remanufactured products droves away some consumers.

## Numerical study

As is shown above, there are two crucial parameters in this study, i.e., the percentage of the tradition demand which is willing to purchase remanufactured products, and the demand coefficient generated by the emission saving. In order to explore the impacts of *β* and *φ* over the decisions, we design three numerical illustrations.

The common parameters are given as follows: the tradition demand quantity *d* = 1000 units, the production cost of a new product *c* = $100, the cost saving of a remanufactured product *s* = $50, the carbon emission of a new product *e* = 100 g, the emission saving of a remanufactured product ∆*e* = 50 g, the carbon tax *λ* = $1, the linear price-sensitive rate *δ* = 2, and the price substitution rate *θ* = 1.

First, we examine the impact of *β* under *β*∈[0.2, 0.8], and set *φ* = 6. In this situation, the given parameters meet Assumption 2. According to (2), the profit for selling new products in a single-product market is *π*_*n*_ = $45000. According to (4), the profit for selling remanufactured products in a single-product market is

$$\pi_{r}=\frac{{\left(1000\beta +100\right)}^{2}}{8}.$$

**Fig 1:**
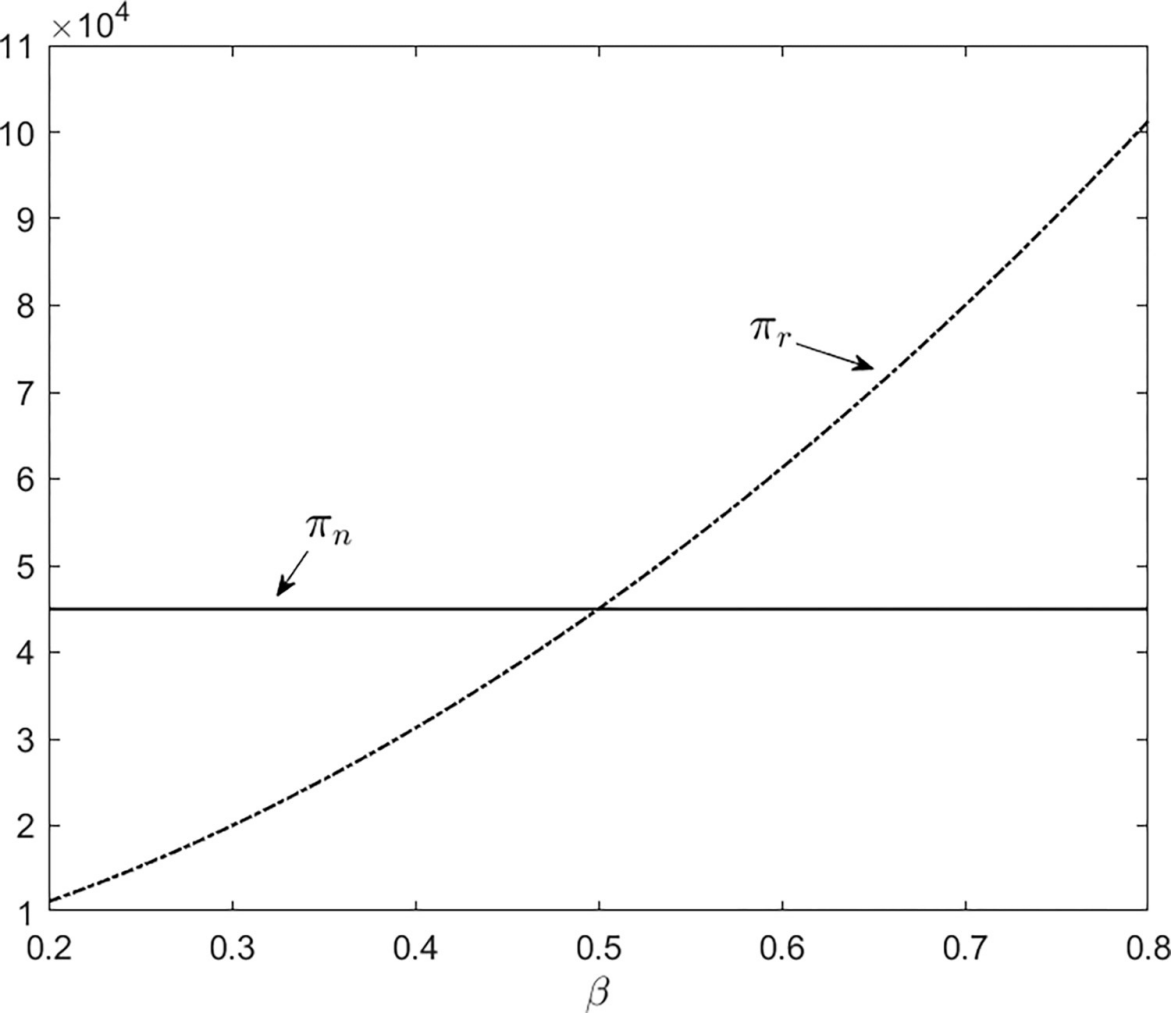
The impact of *β*.

Given the above results, we depict the following curves:

According to Fig 1, *β* is an important factor for the profit of new products. When the value of *β* is low, producing and selling new products is better in the single-product market. Otherwise, producing and selling remanufactured products is a better choice.

Second, we examine the impact of *φ* under *φ*∈[6, 12] and set *β* = 0.4. The profit for selling new products in a single-product market is still *π*_*n*_ = $45000. According to (4), the profit for selling remanufactured products in a single-product market is

$$\pi_{r}=\frac{{\left(200+50\varphi \right)}^{2}}{8}.$$

**Fig 2:**
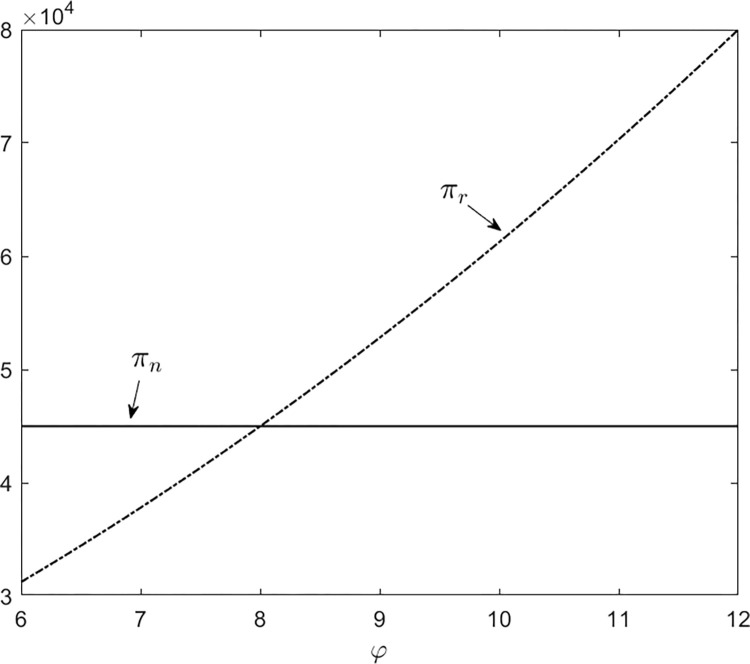
The impact of *φ*.

Given the above results, we depict the following curves:

By Fig 2, it is shown that *φ* has a similar effect on the profit of new products in a single-product market. The difference is that the sensitivity of *φ* is relatively low than the one of *β*.

By the above two examples, we show that *β* and *φ* determine which is the optimal strategy in a single-product market.

Finally, we examine the optimal selling strategy by comparing the three strategies under *β*∈[0.2, 0.8] and *φ*∈[6, 12]. The profit for selling new products in a single-product market remains unchanged. The profit for selling remanufactured products in a single-product market is

$$\pi_{r}=\frac{{\left(1000\beta +50\varphi -200\right)}^{2}}{8}.$$

And the profit for selling both new products and remanufactured products in a mixed-product market is

$$\pi =(1000-3p_{n}+p_{r})(p_{n}-200)+(50\varphi -3p_{r}+p_{n})(p_{r}-100),$$

where

$$p_{n}=\frac{5100+50\varphi }{16},p_{r}=\frac{2500+150\varphi }{16}.$$

**Fig 3:**
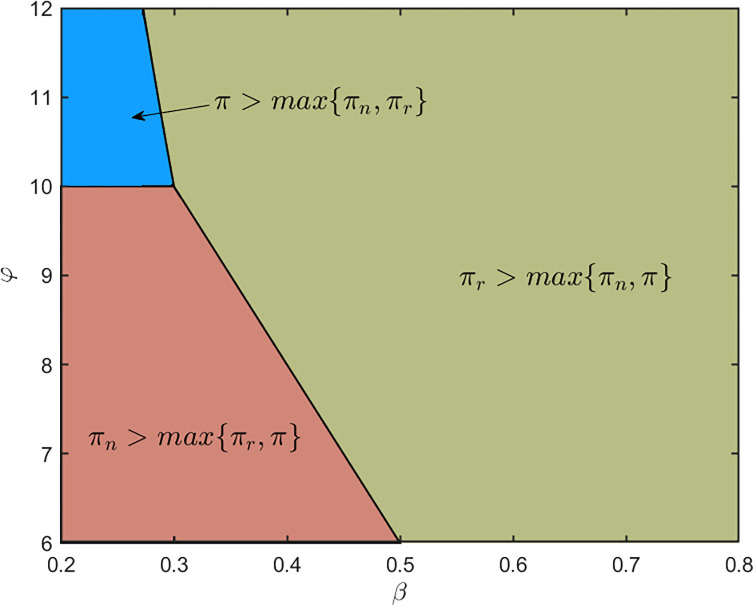
Regions of domination for each strategy.

According to the above results, we present the following picture:

Fig 3 shows the following fact: different from the intuition, the multiple-product strategy (coloured by blue) is not optimal in most region, which is of practical meaning for a manufacturer to formulate production and pricing strategies. Apparently, Suppose 1 is demonstrated by this example.

## Conclusions

This paper proposes pricing models for both the single-product market and the binary-product market in the presence of consumers’ preference. The impacts of crucial factors are explored and three production and pricing strategies are compared so as to analyze the superiority of single-product market and mixed-product market.

Our study draws some managerial insights. By setting an emission sensitive demand, we show the superiority of the remanufactured product when the extra demand attracted by the emission saving is relatively large. In the mixed-product market, the price competition between new products and remanufactured products droves away some consumers. When the extra demand attracted by the emission saving is large and the traditional demand ratio that accepts remanufactured product is high, a single-product market of remanufactured product is optimal.

This paper only considers production and pricing strategies in a single-layer framework. In the further research, it is worth to consider the following aspects: (*a*) the collection process of the waste product; and (*b*) multi-structural framework. The impact of the collection ratio over carbon emission will be revealed by considering these factors.
